# Influence of parental care on offspring hippocampal volume in young adults varies as a function of overprotection

**DOI:** 10.1038/srep46429

**Published:** 2017-04-12

**Authors:** Yinan Wang, Yiying Song, Xueting Li, Lin Zhang, Jia Liu

**Affiliations:** 1 School of Psychology, Beijing Key Laboratory of Applied Experimental Psychology, Beijing Normal University, Beijing, China; 2 State Key Laboratory of Cognitive Neuroscience and Learning & IDG/McGovern Institute for Brain Research, Beijing Normal University, Beijing, China; 3 Department of Psychology, Renmin University of China, Beijing, China; 4 Civil Aviation Medical Center, Civil Aviation Administration of China, Beijing, China

## Abstract

Parental care results in increased hippocampal volumes through adaptive stress responses in developing animals. However, human studies have not yet provided consistent findings analogous to the animal literature, possibly because parental care in humans is likely intermingled with parental overprotection, as suggested by the optimal parenting theory. Here, we tested the hypothesis that the effect of parental care on offspring hippocampal volume varies as a function of parental overprotection with a large cohort of young adult participants (*N* = 257). Consistent with some previous human studies, we found that parental care in childhood alone had little association with the hippocampal volume in adulthood. However, when parental overprotection was low, parental care was positively correlated with offspring hippocampal volume, whereas there was no association between parental care and offspring hippocampal volume when parental overprotection was high. Thus, an interaction exists between parental care and overprotection in human’s hippocampal development, which contributes to the elucidation of the complex relationship between brain structure and environmental factors.

Parenting, including care and protection of the young, is essential for the survival as well as the mental and physical well-being of offspring^1^,^2^. Parenting behaviors are found in diverse forms across a broad taxonomic range^3^ and can exert profound effects on an individual’s brain development. Converging evidence from animal studies shows that better early parenting leads to larger hippocampal volume in the offspring^4^,^5^,^6^,^7^. This facilitation underlies the mechanism through which maternal nurturance promotes adaptive programming of the hypothalamic–pituitary–adrenal axis stress response and hippocampal development^8^,^9^. However, it remains controversial whether the benefits of nurturant parenting revealed in animal studies can extend to humans. A study by Luby *et al*.^10^ revealed that early maternal support predicted later hippocampal volumes, while Rao *et al*.^11^ failed to find any correlation between parental nurturance at a young age and hippocampal volume at adolescence, and even reported a negative relationship between higher-quality parental care at an early age and subsequent hippocampal volumes. Taken together, the relationship between parental care and offspring hippocampal volume identified in animals is likely modulated by other factors in humans.

The theory of optimal parenting^1^ suggests that extreme parental protection, known as overprotection, impedes the positive effects of parental care on the healthy development of the offspring. Follow-up psychosocial studies have shown that offspring with high care and high overprotection report higher distress and lower well-being, as compared to offspring with high care but low overprotection^12^,^13^. Based on current theoretical concepts and empirical evidence indicating that parental care is beneficial to offspring development only when parental protection is not overwhelming, we hypothesize that the beneficial effect of parental care on the offspring hippocampal volume may vary as a function of parental overprotection. In other words, the beneficial effect of parental care on hippocampal volume is present only when parental overprotection is low. Recently, the flexible parenting theory^3^ argued that parents should modify their behavior in response to their offspring’s needs to achieve optimal fitness within the offspring’s development. However, how individuals promote flexible parenting to reach this adaptive goal has rarely been quantified. If an interaction between parental care and overprotection predicts hippocampal development in humans, it will fill a key gap in the literature and lend support to the investigation of complicated mechanisms underlying healthy brain development and important environmental influences like parenting.

To test our hypothesis, a large population of college students was recruited (*N* = 257), and voxel-based morphometry (VBM) was used to examine whether offspring hippocampal volume varies as a function of early parental care and overprotection. Early parental care and overprotection were measured with the Parental Bonding Instrument (PBI)^1^, which is a valid and reliable measure of parenting styles as remembered by participants over 16 years during their first 16 years of life, and consists of two sub-scales (care and overprotection)^14^. We aimed to verify whether early self-reported parental care correlated with subsequent offspring hippocampal volume consistent with the findings of previous studies, and then examined whether parental care showed positive effects on participants’ hippocampal volume only when parental overprotection was low. Besides the hippocampus, we examined whether parental behaviors predicted volumes of the amygdala, hypothalamus, and total white matter, which have been shown to be affected by early experiences, such as childhood poverty^15^ and maternal behavior^16^. Finally, considering that childhood poverty and low parental education can lead to reduction in offspring’s hippocampal volume^15^,^17^,^18^,^19^ and hippocampal volume is found to correlate with one’s self-esteem^20^, we examined whether the interaction of parental care and overprotection was still associated with hippocampal gray matter volume (GMV) after controlling for these potential confounding variables.

## Results

### Correlation between parental care and offspring hippocampal GMV

Means, standard deviations, scoring range, skewness, and kurtosis for all measures are presented in Table 1. The kurtosis and skewness of all the scores ranged from −1 and +1, which indicated the normality of the data. Furthermore, parental care had a low negative correlation with parental overprotection (*r* = −0.27, *p* < 0.001). Similarly, maternal care was negatively correlated with maternal overprotection (*r* = −0.31, *p* < 0.001), and paternal care had negative correlation with paternal overprotection (*r* = −0.19, *p* < 0.01).

To investigate whether parental care was associated with offspring hippocampal volume, we conducted a linear regression analysis that showed parental care was not significantly correlated with hippocampal GMV (*b* = 0.03, standard error (*SE*) = 0.04, *p* = 0.49), when controlling for age, sex, and the total GMV of the whole brain. Thus, early parental care was not correlated with GMV of the hippocampus.

### Parental overprotection moderates the relationship between parental care and offspring hippocampal GMV

To examine whether parental overprotection interacts with parental care on offspring hippocampal GMV, we performed a stepwise multiple regression analysis. The results showed that age had no association with the total hippocampal GMV, while sex was negatively correlated with the total hippocampal GMV (*b* = −0.14, *p* < 0.01), which indicated females had a larger total hippocampal GMV than males. Meanwhile, there was a significant interaction effect between parental care and overprotection on the total hippocampal GMV (*b* = −0.09, *SE* = 0.04, *p* = 0.02, *ΔR*^2^ = 0.009) that showed female participants had larger total hippocampal GMV than male participants. Similar analyses were conducted using the hippocampal GMV in the right and left hemispheres individually. The results of these analyses showed a similar pattern (Table 2).

**Figure 1:**
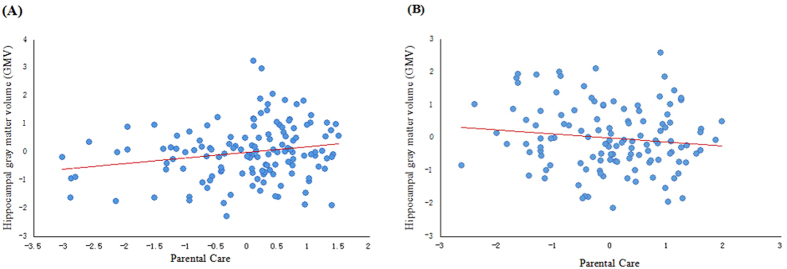
The panel shows scatter plots of correlation between hippocampal GMV and parental care at two levels of parental protection with the median as a cut-off, and the hippocampus GMV was the residue after regressing out age, sex, and total brain GMV as covariates.

To illustrate how parental care and overprotection interacted with each other on hippocampal GMVs, we calculated the partial correlations between parental care and hippocampal GMV for both the low and high parental overprotection groups, after controlling for age, sex, and the total GMV. As our hypothesis predicted, the partial correlation between parental care and hippocampal GMV was significantly positive only for the low overprotection participants (*r* = 0.20, *p* = 0.02, see Fig. 1(A)), but not for the high overprotection participants (*r* = −0.13, *p* = 0.17, see Fig. 1(B)), suggesting that high parental overprotection impeded the positive effects of parental care on offspring hippocampal GMV. When the two outliers (>3 GMV) were removed from the low overprotection participants, the correlation between parental care and hippocampal GMV remains significant (*r* = 0.20, *p* = 0.02).

**Figure 2:**
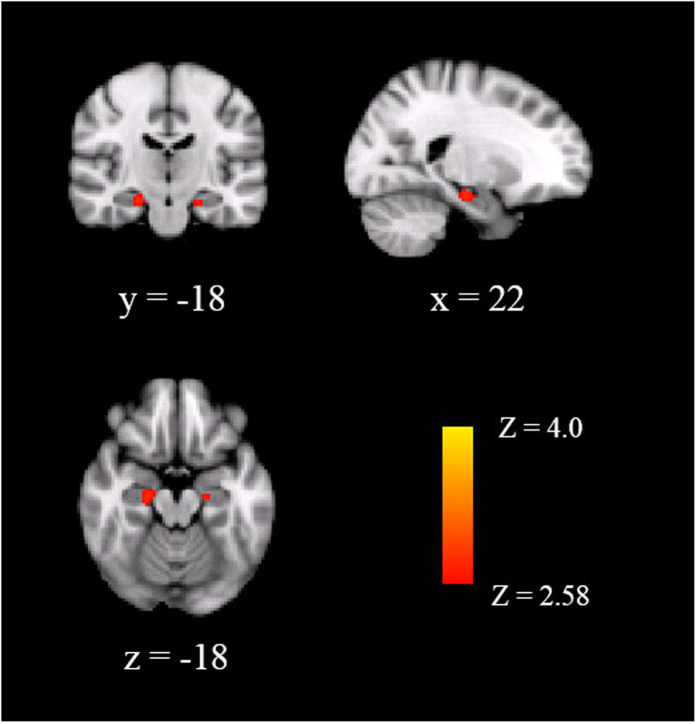
Gray matter volume (GMV) in the hippocampus by region of interest analysis correlated with parental care in low parental overprotection group with age, sex, and total GMV as covariates. The hippocampal GMV was positively correlated with parental care in the group with low parental overprotection. The brain regions were thresholded by a voxel-based threshold of *p* < 0.005, and a cluster-based threshold of *p* < 0.01. The coordinate was shown in the Montreal Neurological Institute stereotactic space.

Additionally, to confirm that parental care correlated with hippocampal GMV in the low parental overprotection group, but not in the high overprotection group, we also performed region of interest (ROI) analysis focused on the hippocampus, separating for the high and low parental overprotection groups. For the low parental overprotection group, the hippocampal ROI analysis revealed a positive and significant correlation between parental care and hippocampal GMV in two clusters of the right and left hippocampus (corrected *p* < 0.005) (see Fig. 2 and Table 3). By contrast, for the high parental overprotection group, hippocampal ROI analysis revealed no significant correlations between GMV of each voxel and parental care. The hippocampal ROI analysis confirmed that parental care was a positive predictor for the offspring hippocampal GMV only when parental overprotection was low.

### The hippocampus was the major brain region influenced by parental behaviors

**Figure 3:**
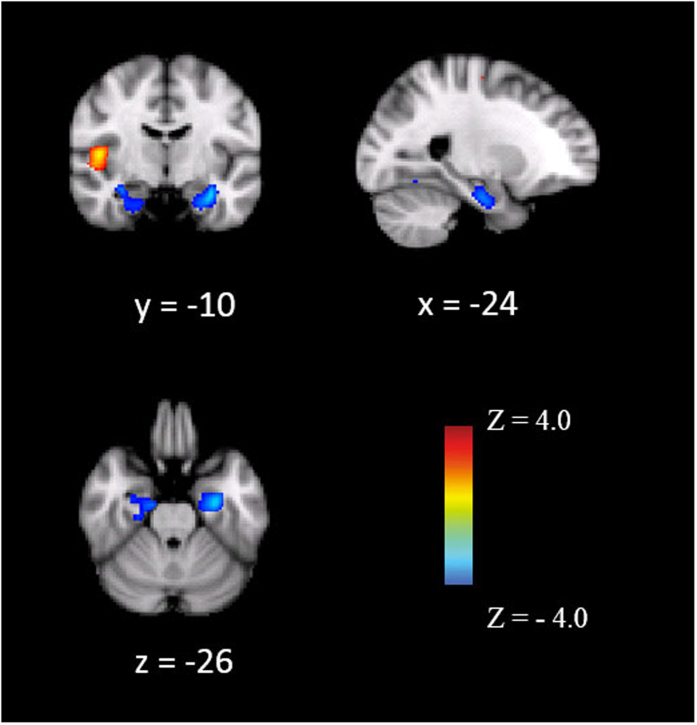
The neuroanatomical correlates with the interaction between parental care and overprotection. A whole-brain exploratory analysis demonstrated that the interaction between parental care and overprotection was negatively associated with increased gray matter volume (GMV) in the bilateral hippocampus and positively associate with increased GMV in the Heschl’s Gyrus at uncorrected (*p* < 0.05) voxel-wise statistical thresholds.

To investigate whether the hippocampus was the primary and major brain region influenced by parental behaviors, we examined whether parental care and its interaction with overprotection predicted GMV of the amygdala and hypothalamus and total white matter volume (WMV). We found that parental care was not significantly correlated with GMV of the amygdala (*b* = 0.02, *SE* = 0.04, *p* = 0.60) or hypothalamus (*b* = −0.001, *SE* = 0.001, *p* = 0.32), or total WMV (*b* = −0.001, *SE* = 0.001, *p* = 0.25), when controlling for age, sex, and the total GMV of the whole brain. Neither was the interaction of parental care by overprotection significantly correlated with GMV of the amygdala (*b* = 0.06, *SE* = 0.03, *p* = 0.08) or hypothalamus (*b* = 0.001, *SE* = 0.001, *p* = 0.98), or total WMV (*b* = −0.001, *SE* = 0.001, *p* = 0.29), when controlling for age, sex, the total GMV of the whole brain, parental care, and parental overprotection. Further, to explore whether the rest of the brain was correlated with the interaction between parental care and overprotection, a whole-brain regression analysis was performed. In addition to the bilateral hippocampus, we found that a significant interaction between parental care and overprotection predicted an increased GMV in Heschl’s Gyrus using an uncorrected *p* < 0.05 voxel-wise statistical threshold (Fig. 3 and Table 4).

### Parental care x overprotection correlated with offspring hippocampal GMV after controlling for confounding variables

We ran five other sets of analyses to ensure these results were reliable. When a similar model was estimated for interaction between maternal care and overprotection, it yielded evidence of a significant interaction effect (Maternal care × Maternal overprotection, *b* = −0.09, *SE* = 0.04, *p* = 0.039, Δ*R*^2^ = 0.007) on hippocampal GMV (see Table 5: Model 1). In a similar pattern, the interaction effect of paternal care and overprotection predicted offspring hippocampal GMV that was marginally significant (Paternal care × Paternal overprotection, *b* = −0.07, *SE* = 0.04, *p* = 0.065, Δ*R*^^2^^ = 0.006) (see Table 5: Model 2). The evidence from maternal and paternal parenting confirmed that the interaction of parental care and overprotection significantly correlated with offspring hippocampal GMV.

Then, subjective childhood socioeconomic status (SES), parental education, and participants’ self-esteem were examined in separate multiple regression models to determine whether they were significantly related to hippocampal GMV. First, subjective childhood SES (*r* = 0.21, *p* = 0.001), parental education (*r* = 0.14, *p* = 0.03), and participants’ self-esteem (*r* = 0.19, *p* = 0.002) were all significantly associated with hippocampal GMV, when controlling for age, sex, and the total GMV of the whole brain. Furthermore, sex had no association with both subjective childhood SES (*r* = 0.08, *p* = 0.21) and self-esteem (*r* = 0.01, *p* = 0.90); however, it had a positive correlation with parental education (*r* = 0.23, *p* < 0.001) that showed males had higher level of parental education than females. Second, none of subjective SES (*b* = −0.01, *p* = 0.89), parental education (*b* = −0.05, *p* = 0.28), or self-esteem (*b* = −0.04, *p* = 0.36) interacted with parental care in predicting hippocampal volume. Hence, we did not include the interaction items of parental care and subjective SES, parental education, or self-esteem in further analyses. At last, multiple regression analysis showed that the interaction of parental care and overprotection was still significantly associated with hippocampal GMV after controlling for subjective SES, parental education, and self-esteem (see Table 5: Model 3,4,5).

Taken together, these results support the hypothesis that the influence of parental care on offspring hippocampal volume varied as a function of overprotection and that early parental care showed positive effects on participants’ hippocampal volumes only when parental overprotection was low.

## Discussion

The present study aimed to investigate whether parental care’s influences on offspring hippocampal volume varied as a function of overprotection in young adults. We observed that self-reported early parental care has no association with offspring hippocampal GMV when the factor of parental overprotection was not considered. However, there was an interaction between parental care and overprotection on human hippocampal development. Specifically, when parental overprotection was low, parental care was positively correlated with offspring hippocampal volume, whereas there was no association between parental care and offspring hippocampal volume when parental overprotection was high. Notably, the interaction effects between parental care and overprotection remained significant even when important confounding factors known to impact hippocampal volume were included in the model. Furthermore, parental care and its interaction with overprotection were not correlated with the volume of amygdala, hypothalamus, and white matter, suggesting the hippocampus was particularly sensitive to parental behaviors. In summary, parental care showed different effects on offspring hippocampal volume depending on parental overprotection in young adults. It was recently proposed that flexible parenting^3^ is critical for the offspring’s healthy development. To our knowledge, our study provides the first neural evidence emphasizing the importance of balancing parental care and protection.

Our finding of no correlation between self-reported parental care with subsequent hippocampal GMV replicates that of some previous studies^11^,^21^,^22^. More importantly, the significant interaction between parental care and overprotection on hippocampal GMV helps explain the inconsistency of the association between parental care and hippocampal volume in humans^10^,^11^. Our findings suggest that early parental care exerts a positive influence on hippocampal volume under the low overprotection condition, but not under the high overprotection condition. One plausible interpretation is that overprotection, which is common or favorable during infancy, has seemingly reversed the positive effects of parental care on subsequent hippocampal volume. In other words, high overprotection may impede the potential benefits of high parental care. The detrimental effect of parental overprotection on offspring might come from its deterrent effect on behavioral autonomy, which is important for human health and well-being^23^,^24^. In contrast, the need for autonomy is likely much weaker in animals than it is in humans; therefore, the association between parental care and hippocampal volume in animals is unlikely to be affected by parental overprotection or control.

The mechanism underlying the interaction of parental care and overprotection on hippocampal volume is unclear. Prior psychosocial studies have shown that parental overprotection is a risk factor for negative health effects by increasing the risk for experiencing depression and distress^25^,^26^. Furthermore, the chronic perceived stress can lead to poor habituation of the hypothalamic–pituitary–adrenal (HPA) axis and dysregulation of glucocorticoid (i.e., cortisol) release^9^. Due to a high density of receptors for glucocorticoids, the hippocampus appears particularly susceptible to damages as a result of chronic stress. And, numerous studies have reported that depressive disorder is associated with smaller hippocampal volumes^27^,^28^,^29^,^30^,^31^. Thus, as a critical neural structure for regulating responses under stress and depression^4^,^32^,^33^,^34^, the hippocampus has been identified to be specifically associated with early parental nurturance^10^,^11^,^15^. Therefore, one possible mechanism is that parental overprotection might result in increased stress and depression for the offspring, which then impedes the positive effect of parental care on the offspring’s hippocampal development. Moreover, the nonsignificant correlation between parental behaviors and the amygdala, hypothalamus, and WMVs supports our stress-related hypothesis about the relationship between parenting behaviors and offspring’s hippocampal development.

Our study extends beyond previous studies by including both mothers and fathers of young adults in our analysis. The similar interaction effect of maternal and paternal care and control on young adults’ hippocampal GMV suggests that fathers play an equally significant role in risk and protective factors for offspring’s hippocampal development. Considering paternal and maternal parenting may have synergistic effects on adolescent outcomes^35^,^36^, future research involving the examination of both triadic (parents and adolescent together) and dyadic (adolescent with each parent separately) interactions will be highly informative^37^.

In addition to parenting, accumulating evidence shows that childhood SES is associated with individual’s hippocampal volume^17^,^18^,^19^. The current study replicated the previous findings that childhood SES is associated with adult’s hippocampal volume^15^,^18^,^38^,^39^. More importantly, we found that the effect of interaction of parental care and overprotection on hippocampal GMV could not be explained by childhood SES. This result suggests childhood material environment (e.g., SES) and psychosocial environment (e.g., parenting) may exert unique effect on individuals’ hippocampal development. Considering that childhood stressful life events (such as trauma, abuse, and maltreatment) will also impair hippocampal development^33^,^40^,^41^, future research is needed to investigate whether early stressful experience will modulate the effect of parenting on offspring’s hippocampal volume.

Two limitations of the current study allow future studies to uncover the exact nature and origin of the association we observed. First, we used a cross-sectional study approach, while aiming to explain changes that are likely to occur because of an interaction between parental care and overprotection. Ideally, longitudinal studies would be required to illustrate how parental overprotection modulates the association between parental care and the hippocampal GMV and to uncover the critical period of psychophysiological development during which the effect of interaction between parental care and overprotection is most pronounced. Another limitation of our design is its dependence on retrospective self-reported measurements of parenting styles, which are susceptible to memory bias and self-preference. Records of parental behaviors that are more objective will help reduce the influence of potential confounding factors.

## Methods

### Participants

Two hundred and fifty-seven college students (112 males; mean age ± standard deviation = 22.65 ± 1.00 year) from Beijing Normal University, China, participated in this study as part of our ongoing project investigating associations among brain imaging, cognitive functions, and genetics^42^,^43^. Participants were instructed to undertake a series of computer-based cognitive ability tests (e.g., reasoning and attention), paper-pencil questionnaires (e.g., family environment and personality), and Magnetic resonance imaging (MRI) scans. Data that were not relevant to the theme of this study are not reported here. Participants reported no past or current psychiatric illness or history of neurological disorders (e.g. epilepsy, traumatic brain injury, neurodegenerative disorders, or cerebro-vascular diseases), no mental retardation, and no significant systemic medical illness. The majority of the participants were right-handed based on a single-item handedness questionnaire (“Are you (a) right-handed, (b) left-handed, (c) ambidextrous?”). The Institutional Review Board of Beijing Normal University approved both the behavioral and MRI protocols and all experiments were performed in accordance with relevant guidelines and regulations. Written informed consent was obtained from all participants prior to the experiment.

### Behavioral Measures

#### Perceived parental care measure

Self-reported parental care was assessed through the subscale of “care” in the PBI^1^. Twelve questions were asked in reference to “care” (e.g., “Spoke to me with a warm and friendly voice” and “Did not help me as much as I needed” (reversed)). The same questions were answered individually for each parent on a four-point Likert scale (0–3). Participants were instructed to answer each question as they remembered their parents in their first 16 years. Both the maternal and paternal composites showed high internal consistency (α = 0.85 and 0.86). We calculated total care scores for both parents by adding maternal and paternal scores (α = 0.91).

#### Perceived parental overprotection measure

Self-reported parental overprotection was assessed using the subscale of “overprotection” in the PBI^1^. Thirteen questions were in reference to “overprotection” (e.g., “Invaded my privacy” or “Let me do those things I liked doing” (reversed)). The same questions were answered individually for each parent on a four-point Likert scale (0–3) (citation). Both the maternal and paternal composites showed high internal consistency (α = 0.85 and 0.83). We created total protection scores for both parents by adding maternal and paternal scores (α = 0.90).

#### Potential confounds

We assessed several demographic, neurobiological, and psychosocial characteristics that might provide alternative explanations for any observed associations, and then modeled them as covariates. The demographic covariates included age and sex. The neurobiological covariate referred to the total GMV of the whole brain. We also included subjective childhood SES and parental education as covariates to control for the possibility that SES influenced both parenting styles and offspring hippocampal volumes^38^,^44^. Participants’ subjective childhood SES was assessed by the youth version of the MacArthur Scale of Subjective Social Status^45^, which measures individuals’ subjective perception of their families’ position on the social ladder as compared to other families in China during their childhood (6–12 years old). Lastly, given that self-esteem has been shown to be positively associated with hippocampal volume^46^, the participant’s self-esteem was treated as psychosocial covariate and was assessed using the Rosenberg Self-esteem Scale^47^. The ten-item scale measures one’s global self-worth, incorporating both positive and negative feelings about the self. The coefficient alpha for the present sample was 0.89, showing a high reliability for the participants to assess their own self-esteem.

### MRI data acquisition

Scanning was conducted on a Siemens 3 T scanner (MAGENTOM Trio, a Tim system) with a 12-channel phased-array head coil at BNU Imaging Center for Brain Research, Beijing, China. A 3D structural MRI was acquired on each subject using a T1-weighted MPRAGE sequence (TR/TE/TI = 2530/3.39/1100 ms, flip angle = 7 degrees, FOV = 256 × 256 mm^2^). One hundred and twenty-eight contiguous sagittal slices were imaged with 1 × 1 mm in-plane resolution and 1.33 mm slice thickness for whole brain coverage.

### Voxel-based morphometry analysis

VBM analysis was employed to quantify gray matter (GM) in each voxel across whole brain^48^. Specifically, VBM was performed using SPM8 (Statistical Parametric Mapping, Wellcome Department of Imaging Neuroscience, London, UK) and DARTEL (Wellcome Department of Imaging Neuroscience). First, image quality was assessed by visual examination. Second, the origin of each brain was manually set to the anterior commissure for each participant. Third, images were segmented into four distinct tissue classes: gray matter (GM), white matter, cerebrospinal fluid and everything else (e.g., skull and scalp) using a unified segmentation approach^49^. Forth, the segmented GM images were rigidly aligned and resampled to 2 × 2 × 2 mm. Fifth, the images were nonlinearly registered with DARTEL, which involves iteratively generating a study-specific template based on the tissue maps from all participants and then warping all participants’ GM images into the generated template to increasingly improve alignment^50^. Sixth, the GM images were normalized into standard MNI space, and the GM voxel values were modulated by multiplying the Jacobian determinants derived from the registration to preserve the volume of tissue from each structure^51^. The modulated GM images were then smoothed with an 8 mm full width at half maximum (FWHM) isotropic Gaussian kernel. Finally, to exclude noisy voxels, the modulated images were masked using absolute masking with a threshold of 0.2. The masked modulated GM images were used for further statistical analyses.

We defined the hippocampus using probabilistic maps from the Harvard-Oxford Subcortical Structural Atlas available for FMRIB Software Library (FSL), including only voxels that had a 50% or greater probability of being labeled as the hippocampus. The total GMV of the right and left hippocampus was calculated by adding the GMV of all voxels within the right and left hippocampal areas, respectively. We also obtained the total hippocampal GMV by adding the hippocampal GMV of both hemispheres. Similarly, we defined the amygdala with the Harvard-Oxford Subcortical Structural Atlas and calculated its GMV. In addition, because prior research has shown that the medial preoptic area of the hypothalamus is influenced by maternal behavior in rodents^16^, we defined the hypothalamic region of interest as a 9 mm radius sphere centered on the Medial preoptic nucleus coordinates (x = 3.5, y = 0.6, z = −13.2) taken from the MRI atlas of the human hypothalamus^52^. Finally, we obtained the total GMV and WMV of the whole brain.

### Statistical Analysis of VBM

First, we investigated whether early parental care was associated with offspring hippocampal volume by conducting a linear regression analysis, with hippocampal GMV as the outcome and with early parental care and covariates (age, sex, and the total GMV of the whole brain) as predictors.

Second, we examined whether the interaction between early parental care and protection predicted the offspring hippocampal GMV. We constructed a regression equation in which hippocampal GMVs were predicted from three successive blocks of variables: the set of covariates, the variables reflecting the main effects of parental care and overprotection, and a product term representing the interaction of the latter two constructs. We determined the statistical significance of all model parameters from the unstandardized estimates calculated on the mean-centered continuous independent variables. Similar analyses were also performed using the hippocampal GMV in the right and left hemispheres as individual dependent variables. Then, to unpack the interaction effect, all participants were split into two groups by the median value of parental overprotection: a high overprotection group (i.e., above the median) and a low overprotection group (i.e., below the median). Then we calculated the partial correlations between parental care and hippocampal GMV after controlling for participants’ age, sex, and the total GMV of the whole brain, for both the low and high parental overprotection groups. To corroborate our results, we also performed a similar regression analysis with the hippocampal masks using SPM8 (Statistical Parametric Mapping, Wellcome Department of Imaging Neuroscience, London, UK).

Third, to confirm that parental care correlated with hippocampal GMV in the low parental overprotection group, but not in the high overprotection group, we performed regression analyses in the ROI of left and right hippocampal areas. Age, sex, and the total GMV of the whole brain were also treated as confounding covariates. Corrections for multiple comparisons were applied using the Gaussian random field theory approach (min *t* > 2.58, cluster significance: *p* < 0.05).

Fourth, to investigate whether the hippocampus was the primary and major brain region influenced by parental behaviors, we examined whether parental care and its interaction with overprotection could predict GMV of the amygdala and hypothalamus and total WMV with similar regression analyses for the hippocampus. Additionally, whole-brain regression analyses were performed by calculating the correlation between GMV of each voxel and the interaction between parental care and overprotection according to a general linear model.

Finally, to ensure that these results were reliable, we examined the interaction between maternal/paternal care and maternal/paternal protection as predicted by the offspring hippocampal GMV. Furthermore, we examined whether the interaction of parental care and parental protection was still significantly associated with hippocampal GMV after controlling for subjective childhood SES, parental education, and participants’ self-esteem as covariates.

## Additional Information

**How to cite this article:** Wang, Y. *et al*. Influence of parental care on offspring hippocampal volume in young adults varies as a function of overprotection. *Sci. Rep.* **7**, 46429; doi: 10.1038/srep46429 (2017).

**Publisher's note:** Springer Nature remains neutral with regard to jurisdictional claims in published maps and institutional affiliations.

## Figures and Tables

**Table 1:** Descriptive statistics and correlations among parental care and overprotection measures.

Scales	Mean	SD	Range	Skewness	Kurtosis	1	2	3	4	5
1. Parental Care	54.10	10.17	24–72	−0.58	−0.07	1				
2. Parental Overprotection	28.48	12.05	0–57	−0.11	−0.67	−0.27***	1			
3. Maternal Care	28.21	5.15	12–36	−0.61	−0.21	0.92***	−0.29***	1		
4. Maternal Overprotection	15.46	6.92	0–33	0.06	−0.46	−0.28***	0.93***	−0.31***	1	
5. Paternal Care	25.89	5.84	5–36	−0.65	0.33	0.94***	−0.22***	0.71***	−21**	1
6. Paternal Overprotection	13.02	6.17	0–28	0.03	−0.74	−0.21**	0.91***	−0.21**	0.69***	−0.19**

*Note*: **p < 0.01 ***p < 0.001.

**Table 2:** Results of multiple regressions of predicting hippocampal GMV.

	Total Hippocampal GMV	Left Hippocampal GMV	Right Hippocampal GMV
Age	−0.02	−0.02	−0.01
Sex	−0.14**	−0.12*	−0.16**
The total GMV of the whole brain	0.65***	0.64***	0.64***
Parental care	0.02	0.01	0.04
Parental overprotection	0.05	0.04	0.07
Parental care x overprotection	−0.09*	−0.10*	−0.08*
*R*^2^	0.58	0.54	0.58

*Note*: *p < 0.05. **p < 0.01 ***p < 0.001.

**Table 3:** GMV in hippocampus by ROI analysis correlated with parental care in low parental overprotection group.

Hippocampal ROIs	Cluster size (voxels)	*p*	MNI coordinate		
			x	y	z
Hippocampus, R	63	0.03	22	−18	−18
Hippocampus, L	16	0.04	−20	−16	−20
Hippocampus, L	10	0.04	−20	−32	−6
Hippocampus, R	4	0.04	18	−28	−8

*Note*: Statistical maps were thresholded at *p* < 0.005 corrected for multiple comparisons. Results were extent threshold corrected at 0.05 at the cluster level.

**Table 4:** Brain regions correlated with the interaction between parental care and overprotection by whole-brain analysis.

Cluster	Cluster size (voxels)	*t* value	MNI coordinate		
			x	y	z
Hippocampus, R	347	4.61	20	−30	−12
Hippocampus, L	306	3.81	−30	−12	−24
Heschl’s Gyrus	541	3.94	48	−10	6

*Note*: Only clusters surviving (*p* < 0.05) for extent and greater than 300 voxels were reported.

**Table 5:** Results of multiple regressions of predicting hippocampal GMV.

Model	*b*	*t*	*p*
Model 1			
Age	−0.01	−0.21	0.83
Sex	−0.15**	−3.08	0.002
The total GMV of the whole brain	0.66***	13.43	<001
Maternal care	0.02	0.39	0.69
Maternal overprotection	0.06	1.39	0.17
Maternal care x overprotection	−0.09*	−2.08	0.039
Model 2			
Age	−0.02	−0.57	0.57
Sex	−0.14**	−2.89	0.004
The total GMV of the whole brain	0.65***	13.21	<0.001
Paternal care	0.03	0.64	0.52
Paternal overprotection	0.02	0.47	0.64
Paternal care x overprotection	−0.07	−1.85	0.065
Model 3			
Age	0.01	0.33	0.743
Sex	−0.15**	−3.14	0.002
The total GMV of the whole brain	0.65***	13.53	<0.001
Subjective childhood SES	0.13**	3.10	0.002
Parental care	0.01	0.15	0.878
Parental overprotection	0.04	0.96	0.337
Parental care x overprotection	−0.09*	−2.17	0.031
Model 4			
Age	0.02	0.43	0.669
Sex	−0.16**	−3.23	0.001
The total GMV of the whole brain	0.65***	13.55	<001
Subjective childhood SES	0.12*	2.60	0.010
Parental education	0.04	0.81	0.421
Parental care	0.001	0.00	1.000
Parental overprotection	0.03	0.80	0.427
Parental care x overprotection	−0.09*	−2.14	0.033
Model 5			
Age	0.02	0.42	0.679
Sex	−0.16**	−3.22	0.001
The total GMV of the whole brain	0.65***	13.62	<0.001
Subjective childhood SES	0.11*	2.36	0.019
Parental education	0.03	0.60	0.556
Self-esteem	0.12**	2.81	0.005
Parental care	−0.04	−0.79	0.431
Parental overprotection	0.05	1.11	0.266
Parental care x overprotection	−0.09*	−2.17	0.031

*Note*: *p < 0.05. **p < 0.01 ***p < 0.001.
